# Recruitment of heterologous substrates by bacterial secretion systems for transkingdom translocation

**DOI:** 10.3389/fcimb.2023.1146000

**Published:** 2023-03-06

**Authors:** Dolores L. Guzmán-Herrador, Andrea Fernández-Gómez, Matxalen Llosa

**Affiliations:** Instituto de Biomedicina y Biotecnología de Cantabria (IBBTEC), Universidad de Cantabria-CSIC-SODERCAN, Santander, Spain

**Keywords:** type III secretion systems, type IV secretion systems (T4SS), type VI secretion systems, heterologous substrate translocation, secretion signals, protein delivery tools, interkingdom protein transfer

## Abstract

Bacterial secretion systems mediate the selective exchange of macromolecules between bacteria and their environment, playing a pivotal role in processes such as horizontal gene transfer or virulence. Among the different families of secretion systems, Type III, IV and VI (T3SS, T4SS and T6SS) share the ability to inject their substrates into human cells, opening up the possibility of using them as customized injectors. For this to happen, it is necessary to understand how substrates are recruited and to be able to engineer secretion signals, so that the transmembrane machineries can recognize and translocate the desired substrates in place of their own. Other factors, such as recruiting proteins, chaperones, and the degree of unfolding required to cross through the secretion channel, may also affect transport. Advances in the knowledge of the secretion mechanism have allowed heterologous substrate engineering to accomplish translocation by T3SS, and to a lesser extent, T4SS and T6SS into human cells. In the case of T4SS, transport of nucleoprotein complexes adds a bonus to its biotechnological potential. Here, we review the current knowledge on substrate recognition by these secretion systems, the many examples of heterologous substrate translocation by engineering of secretion signals, and the current and future biotechnological and biomedical applications derived from this approach.

## 1 Introduction

Bacterial secretion systems (SS) are sophisticated nanomachines that allow bacteria to translocate specific substrates across the cell envelope, so that they can interact with the extracellular media or with other cells. To date, 11 different SS families have been described (T1SS-T11SS (Green and Mecsas, 2016; Bhoite et al., 2019; Gorasia et al., 2020; Grossman et al., 2021; Palmer et al., 2021)), both in Gram-positive and Gram-negative bacteria. The translocated substrates are usually specific proteins, but they can also be nucleoprotein complexes, DNA alone or small molecules, depending on the system. These substrates can be translocated to the extracellular medium or injected into the cytoplasm of a recipient cell, which can be eukaryotic or prokaryotic, so SS can cross up to four membranes.

This versatility to translocate different types of substrates into various final targets is probably the key for the biological success of SS, which are involved in many essential bacterial processes, such as horizontal DNA transfer, motility, mammalian cell infection, or bacterial competition.

Among the 11 families of SS, Type III, IV and VI (T3SS, T4SS and T6SS) have the ability to inject their substrates directly into human cells. Their evolutionary origin, structure, and translocation mechanism are very different. However, all of them are able to deliver effectors to the cytoplasm of mammalian cells, with the aim of subverting the cells for the benefit of the bacteria (Galán and Waksman, 2018; Bleves et al., 2020). This capability to act as transkingdom injectors endows them with the potential to serve many biotechnological and biomedical applications if they can be tailored to secrete the substrate of our choice. In this review we will focus on effector recruitment, with emphasis on the sequences or domains present in the substrates that allow their recruitment and subsequent translocation. We will describe numerous examples of adding these recruitment signals to heterologous proteins, in order to make them recognizable by SS and translocatable to recipient cells. In fact, the use of these SS as universal injectors already includes some cases of biotechnological or biomedical applications, and the new advances in understanding secretion signals are likely to increase their potential uses in the near future.

## 2 Biological function of bacterial secretion systems

### 2.1 Type III secretion systems

T3SS are multiprotein complexes encoded by Gram-negative bacteria, which allow the translocation of proteins across the bacterial membranes. T3SS can be found in two very different structures evolved from a common ancestor: the injectisome and the flagellum (Diepold and Armitage, 2015). In the flagella, the majority of the components are exported by the T3SS for its construction, while the injectisomes translocate proteins into eukaryotic host cells, and will be the focus of this review. Injectisomes are mainly encoded by animal or plant pathogens such as *Pseudomonas, Salmonella, Shigella* or *Yersinia*, but also in symbionts like *Rhizobium* spp. (Coburn et al., 2007; Horna and Ruiz, 2021). These systems are involved in a variety of activities that require close interaction between the bacteria and the host cells, such as the modulation of the actin cytoskeleton for cell invasion, prevention of phagocytosis, interfering with immune response or promoting nodule formation (Sory and Cornelis, 1994; Jarvis et al., 1995; Marketon et al., 2005; Shaw et al., 2005; Holmes et al., 2010; Mou et al., 2018). Translocation of the substrates (generally called effectors) through the T3SS is essential for the virulence of many different pathogens. The number of effector proteins translocated varies depending on the pathogen; there are bacteria like *Pseudomonas* which transfer a few effectors, while others like enterohemorrhagic *Escherichia coli* (EHEC) translocate dozens (Larzábal et al., 2018; Horna and Ruiz, 2021).

### 2.2 Type IV secretion systems

T4SS are multiprotein complexes that span bacterial membranes in both, Gram-negative and Gram-positive bacteria. They are nanomachines with high plasticity, as they can translocate both DNA and proteins to the milieu or into another cell, either prokaryotic or eukaryotic. T4SS can be divided into three subfamilies, according to their biological function: conjugation, DNA export and import, and protein translocation to recipient cells (Grohmann et al., 2018; Li et al., 2019). The first ones are specialized in the horizontal transfer of DNA between bacteria, contributing to the dissemination of antibiotic resistances (Cabezon et al., 2015; Koraimann, 2018). The translocated substrate is a protein (known as the relaxase) covalently linked to a single-stranded DNA molecule, which is injected directly into the cytoplasm of the recipient bacteria. The VirB/D4 T4SS of *Agrobacterium tumefaciens* is of particular interest since, in this case, the nucleoprotein complex is transferred into plant cells.

The second type of T4SS mediates DNA intake and secretion between the bacteria and the medium, and is involved in activities such as DNA exchange with the medium or biofilm formation (Hofreuter et al., 2001; Pachulec et al., 2014). Lastly, T4SS that translocate proteins into the recipient cells participate in two very different biological functions. Most are involved in pathogenesis, since they are encoded by bacterial pathogens such as *Legionella pneumophila, Salmonella enterica* or *Xantomonas citri*, and they translocate effector proteins into the eukaryotic cells targeted by these pathogens. However, T4SS can also mediate symbiotic relationships, such as in the case of *Synorhizobium meliloti* and legumes (Nelson et al., 2017). A subfamily of these protein secretors, however, are involved in toxin secretion to other bacteria, playing a role in bacterial competition (Souza et al., 2015).

### 2.3 Type VI secretion systems

Bacterial T6SS are protein translocating nanomachines present in many different Gram-negative bacteria. Their structural components share homology with the contractile tails of phages (Leiman et al., 2009). In fact, they deliver a wide variety of effector proteins into target cells using a contraction-based mechanism similar to that of bacteriophages. T6SS are involved in different processes, depending on the translocated protein and the target cell. Originally, they were described as “bacteria killing weapons”, since they injected antibacterial effector proteins into surrounding sensitive bacteria (Hood et al., 2010; Russell et al., 2011; Russell et al., 2013; Ma et al., 2014; Whitney et al., 2015; Pissaridou et al., 2018). Remarkably, effectors with antibacterial activities are encoded together with a cognate immunity proteins, which prevents self-intoxication acting as antitoxins (Russell et al., 2012). It was then discovered that T6SS can also play a role in eukaryotic cell targeting, as is the case of T6SS of *Vibrio cholerae* or the H2-T6SS from *Pseudomonas aeruginosa* (Hachani et al., 2016; Monjarás Feria and Valvano, 2020). Interestingly, T6SS can translocate similar toxins to both prokaryotic and eukaryotic cells (Berni et al., 2019). At present, they have been associated with other activities, such as the release of metal-scavenging proteins into the media to provide metals to the bacteria (Wang et al., 2015; Si et al., 2017a; Si et al., 2017b; Han et al., 2019). The different effector molecules and their specific activities are reviewed in Hernandez et al., 2020; Monjarás Feria and Valvano, 2020 and Jurėnas and Journet, 2021.

## 3 Structure, substrate recruitment and secretion mechanism

### 3.1 Type III secretion systems

Despite the variety of substrates and activities in which these systems are involved, their structure is highly conserved (Wagner et al., 2018; Jenkins et al., 2022). Since the T3SS of *Salmonella enterica* serovar Typhimurium was first isolated and imaged (Kubori et al., 1998), the improvement of techniques such as cryoelectron microscopy, cryoelectron tomography or single-particle analysis has helped define precisely the structure of these systems (Abrusci et al., 2012; Hu et al., 2019; Bergeron and Marlovits, 2022; Jenkins et al., 2022). The injectisome crosses the inner and outer membrane (IM and OM) of Gram-negative bacteria and extends into the eukaryotic cell cytoplasm generating a continuous channel which resembles a nanomolecular syringe and allows the delivery of the effector proteins. The needle complex contains the syringe-like structure that spans both bacterial membranes and is involved in the transport of the effectors. It is anchored to the cell envelope through a multi-ring base, the basal body. The injectisome is composed by a highly conserved export apparatus which forms a channel crossing the IM, and the needle, formed by a single protein which polymerizes helically and expands into the extracellular milieu. The needle complex is connected with the cytoplasm of the bacteria through the sorting platform, composed of soluble proteins that regulate substrate secretion in association with a central ATPase, which seems to play a role in the unfolding of the effector proteins and their dissociation from the chaperones (Akeda and Galán, 2005; Minamino et al., 2014).

T3SS secrete a wide variety of substrate proteins across both the IM and OM. Usually, the mechanism is a one-step process in which the bacteria inject effector proteins through a pore formed by a structure, known as translocon, in the target cell after host cell contact. However, other models have also been proposed, since some T3SS have been found in which the secretion of effectors and the translocon complex occurs before contact with the target cell, and they remain associated to the bacteria until they are translocated when contact takes place (Edgren et al., 2012). Moreover, heterologous substrates, fused to the translocation signals (TS), are detected in the culture supernatant prior to their detection in eukaryotic cells, which allows measuring the secretion rates (Rüssmann et al., 1998; Crawford et al., 2002; Chen et al., 2006; Nishikawa et al., 2006; Widmaier et al., 2009; Carleton et al., 2013; Metcalf et al., 2014).

T3SS substrates must be guided to the system and introduced in the secretion channel. For this to happen, T3SS substrates have a secretion signal at the N-terminus and most of them also a chaperone-binding domain (CBD) downstream (see below). The latter allows chaperones to bind the substrate, forming the chaperone-substrate complex in order to maintain the effector partially unfolded and guide it to the cytoplasmic components of the T3SS (Stebbins and Galán, 2001; Wagner et al., 2018). The chaperone-substrate complex interacts with members of the sorting platform and is guided to the base of the injectisome (Diepold et al., 2017). It is proposed that the translocation of the substrate occurs in a polarized fashion (N-terminal region of the substrate enters first) through the needle (Radics et al., 2013; Miletic et al., 2021) and the EA functions as a gate and a guide to transport the substrate to the filament (Miletic et al., 2021).

Effector translocation occurs rapidly and in a hierarchical manner (Schlumberger et al., 2005; Lara-Tejero et al., 2011). One important point of this process is the unfolding of the substrate. The inner diameter of the injectisome needle is 25-35 Å (Loquet et al., 2012), and therefore the T3SS substrates must be, at least, partially unfolded to cross the tube. The unfolding process is complex, involving the already mentioned chaperones, an ATPase involved in the stability of the protein, the effector folding-unfolding kinetics or the proton motif force, which are also essential during the translocation of the substrates (Akeda and Galán, 2005; Wilharm et al., 2007; Dawson et al., 2009; Khanppnavar et al., 2020). Moreover, it has been shown that translocation through the T3SS is limited to specific substrates with certain characteristics, and proteins which have a very stable structure or are rapidly folded cannot be translocated. This is the case of the green fluorescent protein (GFP) or ubiquitin (Feldman et al., 2002; Lee and Schneewind, 2002; Akeda and Galán, 2005; Sorg et al., 2005; Riordan et al., 2008; Dohlich et al., 2014). Recently, it has been suggested that the T3SS unfolding process is a mechanical and labile process which facilitates the efficient secretion of the effectors (LeBlanc et al., 2021). Since effectors are unfolded while in bacteria, they are assumed to be inactive in them. However, some exceptions have been found; the orthologue effectors NleB/SseK from *Citrobacter rodentium, S*. Typhimurium and *E. coli* have been reported to be active in the bacteria before their translocation, where they seem to play an important physiological regulator role and improve bacterial survival under unfavourable environmental conditions (El Qaidi et al., 2020; El Qaidi et al., 2021; Xue et al., 2022).

As previously mentioned, effectors translocated through T3SS generally contain an N-terminal secretion signal, a CBD and a C-terminal effector domain. For many substrates, both the N-terminal signal and the CBD are essential for T3SS recognition, recruiting and secretion (Lee and Galán, 2004). For example, deletion of the CBD site in some effectors results in abolition of their translocation through their cognate T3SS. This was demonstrated with SopE and SptP effectors from the T3SS encoded in *Salmonella* pathogenicity island 1 (SPI1) (Lee and Galán, 2004). In this work, they proved that the deletion of the CBD of these effectors resulted in their secretion through a flagellar T3SS instead of their specific T3SS. The authors suggest the existence of an ‘ancestral’ T3SS flagellar secretion signal that is revealed in the absence of the CBD. However, in many other cases the N-terminal signal has been found to be enough for secretion of the effectors or other reporter proteins fused to these sequences (Anderson and Schneewind, 1997; Crawford et al., 2002; Rüssmann et al., 2003; Charpentier and Oswald, 2004; Cardenal-Muñoz and Ramos-Morales, 2011). In the case of YopE of *Yersinia*, either the N-terminal signal or the CBD were sufficient for the secretion of the effector or other heterologous substrates fused to these tags (Cheng et al., 1997).

The N-terminal signal was elusive for a long time and fed a debate that is still open (Hui et al., 2021). Some authors proposed that the signal could be at the mRNA level, since frameshift mutations in the sequence of some effectors were tolerated (Anderson and Schneewind, 1997; Anderson and Schneewind, 1999). However, the main accepted idea is that the TS are in the N-terminus of the secreted peptides (Michiels and Cornelis, 1991; Sory and Cornelis, 1994; Karavolos et al., 2005). The signal comprises a very variable sequence of 10-25 residues, showing no homology even between effectors translocated through the same T3SS, as happens with *Yersinia* Yop effector proteins (Michiels and Cornelis, 1991). Despite this heterogeneity, usually they contain mainly polar and not charged or hydrophobic amino acids, producing a lack of secondary structure. In fact, it is possible to predict T3SS effector proteins from different bacteria as these N-terminal sequences have similar characteristics (Arnold et al., 2009; Samudrala et al., 2009). Sequence variability could also make N-terminal signals quite tolerant to mutations.

There are some well-defined TS. It is the case, for instance, of YopE and YopH from *Yersinia enterocolitica* (Sory and Cornelis, 1994), where the N-terminal 15 and 17 amino acids respectively are needed. For ExoS from *P. aeruginosa*, the TS comprises the 54 N-terminal amino acids (Epaulard et al., 2006), and for SopE from *S.* Typhimurium, the 60 N-terminal residues are required (Karavolos et al., 2005).

The CBD, a ca.50-100-amino-acid domain, is found downstream the secretion signal (Akeda and Galán, 2005; Rodgers et al., 2008; Tsai et al., 2015). Chaperone binding to the CBD serves many functions: they stabilize the substrate in the bacterial cytoplasm, prevent the erroneous targeting of the effector or maintain the substrate in a partially unfolded state, which is necessary for the subsequent translocation (Stebbins and Galán, 2001; Lara-Tejero et al., 2011; Krampen et al., 2018; LeBlanc et al., 2021). The involvement of chaperones in T3SS substrate translocation was observed for the first time in *Y. enterocolitica.* Four different chaperones were described specifically for the translocation of *Yersinia* effectors (Yops) (Wattiau and Cornells, 1993; Wattiau et al., 1996).

### 3.2 Type IV secretion systems

In the last few years, great advances have been reported in the definition of T4SS architectures and assembly pathways, such as the ones from the pathogens *L. pneumophila* and *Helicobacter pylori*, or the conjugative plasmids R388 and F (Durie et al., 2020; Liu et al., 2022; Macé et al., 2022). In Gram-negative bacteria, almost all T4SS share a conserved structure composed of 12 subunits (Costa et al., 2021; Sheedlo et al., 2022), named VirB1-VirB11 and VirD4, as it was established from the paradigmatic *A. tumefaciens* VirB/D4 T4SS. The structure can be divided into the extracellular pilus, the core complex spanning both bacterial membranes, and the cytoplasmic ATPases, located at the base of the translocation channel, that supply energy to the system. Among them, VirD4, also known as the coupling protein (CP), plays an important role in the recruitment of substrates and their translocation though the IM (Macé et al., 2022).

T4SS can recruit and translocate an arsenal of substrates by the recognition of different TS and accessory adapter proteins or chaperones bound to the substrates. In the case of T4SS involved in conjugation, the relaxosome is the complex recruited by the T4SS. It is formed by the relaxase, the *oriT* containing DNA and other accessory proteins. The actual substrate of the system is the relaxase (Guzmán-Herrador and Llosa, 2019), which is translocated into the recipient covalently linked to the transferred DNA strand. On the other hand, effector translocation systems deliver proteins into the target cells which interfere with their functions. The number of effectors is very variable, as there are systems like *L. pneumophila* Dot/Icm T4SS that can translocate hundreds of effectors, while others like *H. pylori* Cag T4SS encode only one (Backert et al., 2010; Du Toit, 2019). In the last decade, some T4SS have been reported to secrete antibacterial effectors which target the surrounding bacteria, such as X-T4SS (Xanthomonadales-like T4SSs) in *Xanthomonas* spp. or *Stenotrophomonas* spp. (Souza et al., 2015; Bayer-Santos et al., 2019; Sgro et al., 2019).

The pathway followed by the substrate and its interactions with the different elements of the system were first studied by an assay termed TrIP (Transfer DNA ImmunoPrecipitation), which determined the close sequential contacts between the transferred DNA and T4SS proteins in the VirB/D4 T4SS from *A. tumefaciens* (Atmakuri et al., 2004; Cascales and Christie, 2004). The substrate was found to interact first with cytoplasmic ATPases, then inner membrane complex proteins, outer membrane core complex, and, eventually, VirB2 in the pilus. However, this model does not fully fit with the known structure of T4SS (Waksman, 2019). One of the key elements of the translocation process are the type IV coupling proteins (T4CP), ATPases believed to be involved in substrate recruiting for its subsequent secretion and delivery through the rest of the T4SS. The interaction between the T4CP and the substrate is still not clear. In some systems, a direct interaction between the substrate and the T4CP has been reported, as happens with *X. citris* effectors, VirD2 from *A. tumefaciens*, *Enterococcus faecalis* PcfC or TrwC from R388 (Llosa et al., 2003; Whitaker et al., 2015; Oka et al., 2022). In other cases, additional proteins are also implicated, as happens with Cag proteins in *H. pylori* Cag T4SS, with the Type IV Coupling Complex (T4CC) in *Legionella* Dot/Icm, with the “recruiting proteins” described in *A. tumefaciens*, or with other proteins from the relaxosome such as TraM from F plasmid or TrwA from R388 plasmid (Llosa et al., 2003; Guo et al., 2007; Jurik et al., 2010; Kim et al., 2020; Meir et al., 2020).

As happened with T3SS effectors, the unfolding of the T4SS substrate is a prerequisite for its translocation through the complex. This unfolding process has been studied by fusing proteins that are very stable or known to fold rapidly, like GFP, ubiquitin or dihydrofolate reductase (DHFR) to the different T4SS substrates and determining how the translocation was abolished or reduced. This unfolding process is required for effector proteins like the ones in *L. pneumophila* or *H. pylori* Amyot et al, 2013; Lettl et al., 2021), and also conjugative relaxases (Trokter and Waksman, 2018).

T4SS substrates carry a TS in order to be recognised and translocated to the recipient cell, whose localization varies depending on the translocated substrate. Effector proteins usually carry this TS at the C-terminal region. In *Coxiella burnetii* for example, it has been proven that the 50 C-terminal residues of an effector were necessary for its translocation, since the deletion of the sequence abolishes this delivery, which is rescued when the TS is recovered (Chen et al., 2010). Similar results were obtained for *L. pneumophila* effectors (Nagai et al., 2005). Although there are few works related to the prediction of T4SS effectors by their C-terminal region (McDermott et al., 2011; Wang et al., 2014), the variability among TS is ample, and there is no consensus sequence for all the T4SS effectors. Two types of TS were originally identified, one composed of clusters of positively charged residues and a second composed of hydrophobic residues. For example, the C-termini of the different effectors that are translocated through *A. tumefaciens* VirB/D4 have shown a consensus Arg motif, and the importance of these positively charged residues was proven in effector VirF (Vergunst et al., 2005). However, in other effectors, very different TS have been described, as in the effectors of the bacterial-killing T4SS of *X. citris* (X-T4SS), with antibacterial activity. These effectors carry a specific C-terminal domain called XVIPCD (*Xanthomonas* VirD4 interacting protein conserved domain), a sequence of ca.120 residues required for its interaction with the T4CP VirD4 and for translocation. XVIPCDs are characterized by several conserved motifs and a glutamine-rich tail (Alegria et al., 2005; Souza et al., 2015; Bayer-Santos et al., 2019; Oka et al., 2022). Bioinformatic analysis showed that they were present in many different species of bacteria, not only Xanthomonadales, but also other proteobacteria (Sgro et al., 2019) and, in fact, it has been reported that this motif is necessary for the recruitment of an effector from the X-T4SS of *Stenotrophomonas maltophilia* (Bayer-Santos et al., 2019).

Some effectors carry bipartite TS. For instance, the C-terminal region of the CagA effector from *H. pylori* T4SS is required but not sufficient for its translocation, also requiring an N-terminal region of 20 amino acids, adjacent to a larger CBD necessary for secretion (Hohlfeld et al., 2006). The domain known as BID (Bep intracellular delivery) was discovered in the *Bartonella* effector proteins (Bep), which encoded the positively charged tail sequence and the proximal BID domain. This domain was shown to be present not only in *Bartonella henselae* effectors (in some cases in more than a copy), but also in other species of the genera. Surprisingly, the BID domains were also detected in conjugative relaxases. One of them, from the AvhB/TraG conjugative system from a plasmid of *Agrobacterium*, was recognised and translocated through the VirB/D4 from *B. henselae* to a eukaryotic cell (Schulein et al., 2005).

*L. pneumophila* Dot/Icm T4SS translocates hundreds of effector proteins. The first TS of these effectors was described as the typical C-terminal signal which was enriched in small, polar and charged amino acids (Nagai et al., 2005; Kubori et al., 2008; Burstein et al., 2009). Later, a motif termed “E-block motif” was identified in more than 100 *L. pneumophila* effectors, which was rich in Glu residues and located at the C-terminus (Huang et al., 2011). Besides the C-terminal TS, the translocation of *L. pneumophila* effectors can be also affected by the IcmS-IcmW chaperones (Ninio et al., 2005; Cambronne and Roy, 2007; Burstein et al., 2016). It has been described that the translocation of many effectors can be modulated by these chaperones; while for some of them in the absence of the chaperones the level of translocation is drastically reduced (IcmSW-dependent effectors), other effectors are not affected (IcmSW-independent effectors). Taken all these facts into account, it has been proposed that *Legionella* effectors can be recruited in three different ways: (i) *via* the E-block motif; (ii) effectors that use both the C-terminal secretion signal and the IcmS–IcmW chaperon complex for translocation; and (iii) effectors that use mainly the IcmS–IcmW chaperon complex for translocation (Lifshitz et al., 2013). Another motif has been found in the C-terminal region of some effectors, but not all of them, that allows its interaction with an adaptor protein, suggesting the existence of more binding motifs or the possibility of other proteins working as adaptors (Kim et al., 2020).

In the case of T4SS involved in conjugation, the substrates are the relaxase proteins. The TS carried by the relaxases are different and usually larger than the previously discussed. Several of them have been mapped, among which are the following: MobA from R1162 carries two different internal signals in positions 204-323 and 322-387 (Parker and Meyer, 2007); TraI from the F plasmid and R1 TS are located at 530-816 and 1255-1564 (Lang et al., 2010); and TrwC from R388 has one TS located at 796-802, which is conserved in the relaxase TraI from pKM101, closely related to TrwC (Alperi et al., 2013). There are several relaxases which can be translocated through different types of T4SS. In this case, interestingly, it has been reported that relaxases may carry different translocation signals for recognition by different T4SS. MobA can be recruited by conjugative T4SS through its internal TSs, and is recruited by *A. tumefaciens* VirB/D4 by its C-terminal 48 amino acids, like Vir effectors (Vergunst et al., 2005). Similarly, in the relaxase TrwC, which can be recognized by its cognate R388 T4SS and by the VirB/D4 T4SS of *B. henselae* (Fernández-González et al., 2011), the TS which drastically reduces conjugative transfer when mutated, did not affect TrwC recruitment by VirB/D4 T4SS. Conversely, changes in the C-terminal end of TrwC affected the translocation through *B. henselae* VirB/D4, but did not alter conjugation frequencies (Alperi et al., 2013).

### 3.3 Type VI secretion systems

T6SS deliver effectors into target cells using a spring-like mechanism, so that they have two different conformations: extended and contracted, and operate *via* cycles of assembly-contraction and disassembly (Cherrak et al., 2019). The system is formed by a puncture structure, the tail, which contains a tube of Hcp proteins, a contractile sheath which wraps the tube, and a distal spike or spike complex formed by VgrG and PAAR proteins. All these elements form the tail tube/sheath complex. The system is anchored to the cell envelope by the membrane complex, which also serves as a docking platform for the cytoplasmic baseplate structure. The baseplate connects the membrane complex with the tail tube/sheath complex and is where the polymerization of both, the tube and the sheath, is initiated.

The T6SS substrates differ from the ones of T3SS and T4SS in that they are proteins associated with elements of the needle (Hcp, VgrG and PAAR). Upon sheath contraction, the Hcp tube, together with the spike formed by VgrG and PAAR are propelled into the milieu or into the target cell, and substrates are sent along with them.

There are two types of T6SS effectors: cargo and specialized or evolved. Cargo effectors interact non-covalently with the Hcp, VgrG and PAAR proteins from the needle structure. These interactions can be assisted by chaperones or due to structural motifs. Specialized or evolved effectors contain an N-terminal domain of a structural component (Hcp, VgrG or PAAR), essential for the T6SS assembly, and a C-terminal domain that consists of an extension with an effector domain. The delivery mechanism is different depending on the type of effector or the interacting component (Cherrak et al., 2019; Hernandez et al., 2020; Jurėnas and Journet, 2021). Its localization is determined by the component which it is associated with. Effectors that are fused or interact with PAAR or VgrG are part of the puncturing structure (Flaugnatti et al., 2016; Rigard et al., 2016), while the ones associated with Hcp are inside the tube. Therefore, large effectors such as phospholipases are usually delivered through VgrG associations, since they do not fit inside the tube, while smaller peptides use to be loaded inside the lumen of the Hcp tube (Mougous et al., 2006; Russell et al., 2013; Silverman et al., 2013; Wettstadt et al., 2019; Flaugnatti et al., 2020). The effector proteins which interact with Hcp and therefore are inside the tube have been suggested to be in an unfolded state, as they showed instability in the absence of Hcp, suggesting that they do not adopt their final folded conformation until they are released from the Hcp pore (Silverman et al., 2013). Effectors that interact with other components are loaded in a folded state.

Different cargo and specialized effectors have been found associated with all three components, Hcp, PAAR and VgrG. Effectors associated with Hcp are inside the tube, while VgrG and PAAR associated effectors are believed to be located outside the spike. The number of effectors found varies depending on the protein it is associated with and the type of effector. For example, while the VgrG or PAAR specialized effectors seem to be very extended (Pukatzki et al., 2007; Ma et al., 2009; Koskiniemi et al., 2013; Shneider et al., 2013; Toesca et al., 2014; Sana et al., 2015; Rigard et al., 2016; Ma et al., 2017b), the Hcp are less spread (Ma et al., 2017a).

Cargo effectors may also need the action of specific chaperones or adapters, which are usually encoded next to their cognate effector and are indispensable and specific for the substrate translocation, although they are not secreted with them. They have functions related to the folding of the effector, but their main function is related to recruitment, as they are going to load the specific effector on the correct T6SS element. Different families exist depending on the protein which interacts with the chaperone, and they carry specific domains (see Manera et al., 2021 for review).

Some VgrG or PAAR proteins contain C-terminal extension domains involved in direct interactions with effectors. They participate in several functions as substrate recruitment, substrate stabilization or effector neutralization, among others (Bondage et al., 2016; Flaugnatti et al., 2020). It was found that some substrates from T6SS carry an N-terminal conserved motif extended among different effectors and putative effectors. This sequence was named MIX (Marker for type sIX effectors) (Salomon et al., 2014). MIX sequences were classified in five clans due to the considerable diversity that they showed (MIX I - MIX V). Generally, they contain a conserved central motif hRxGhhYhh (where h represents hydrophobic residues) and two less conserved motifs at the C-terminus and at the N-terminus (shhPhR and hhF/YSxxxWS/T respectively). MIX appears to be mainly located at the N-terminal region of the proteins, fused to C-terminal effector domains with antibacterial or anti-eukaryotic activity (Salomon et al., 2014). A recent work has shown that MIX sequence is necessary for the translocation of the effector from T6SS1 of *Vibrio parahaemolyticus*, demonstrating the importance of this signal in substrate recruitment (Fridman et al., 2022). Recently, it has been proposed that these sequences can be found not only in the effector protein, but also in a co-effector, which enables the loading and secretion of the toxin *via* the T6SS (Dar et al., 2022). However, MIX sequences are not the only secretion motifs found. Jana and collaborators reported that the N-terminal domain of a toxin delivered by the T6SS1of *V. parahaemolyticus* was necessary for the translocation of the effector, since its deletion prevented translocation. Using informatic analysis they found that this domain was extended among different T6SS effector proteins and it was called FIX (Found in type sIX effector) (Jana et al., 2019). These FIX sequences consist of a ca.80 amino acids sequence located at the N-terminus region of the protein, and they are usually fused to the C-terminal region of toxin domains. These sequences have been also found in effectors which contain an N-terminal VgrG or PAAR domain. Both, MIX and FIX sequences seem to be mutually exclusive as, so far, no proteins containing both sequences have been found.

## 4 Heterologous substrate translocation into target cells

### 4.1 Translocation through T3SS

**Figure 1:**
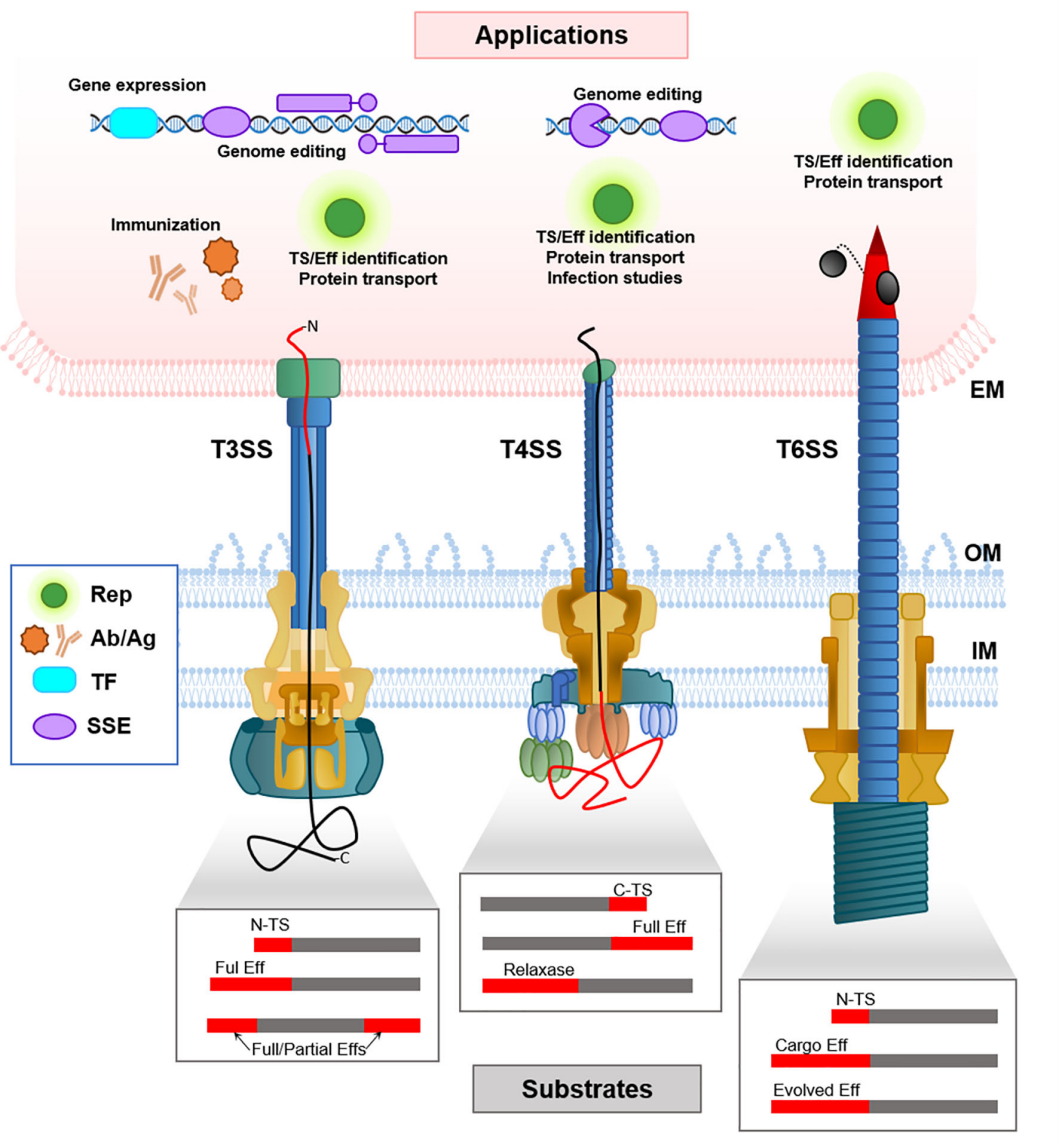
Schematic representation of the delivery mechanisms of heterologous substrates mediated by T3SS, T4SS and T6SS (bottom part), and the applications in the target eukaryotic cell (upper part). Note that in T4SS it is not known if the N- or C-terminus of the translocated peptide enters first. The boxes schematize the different kinds of fusion proteins that have been generated in order to translocate heterologous substrates through each SS, where the heterologous substrate is shown in grey, and the N-terminal or C-terminal translocation signals (N-TS, C-TS), or the full effector (Eff) are shown in red. EM, eukaryotic membrane; IM, Inner Membrane; OM, Outer Membrane; TS, Translocation Signal; Rep, Reporter proteins; Ab/Ag, Antibodies and antigens; TF, Transcription factors; SSE, site-specific endonucleases.

There are several studies on the use of secretion signals from T3SS effectors to deliver heterologous proteins into the cytoplasm of target cells or the extracellular medium (**Table 1**). The heterologous substrates were in most cases reporter proteins which allowed detection and measurement of effector translocation. To achieve this translocation, the protein can be fused either to a tag present in the effector or to the native effector itself **(** **Figure 1** **)**.

**Table 1:** Compilation of the heterologous substrates that have been translocated into eukaryotic cells by the bacterial T3SS, T4SS and T6SSs, indicating the bacteria from which they have been delivered and the fusions with effectors generated.

Delivery strain	Secretion system	Heterologous substrate	Effector fusion	Target cell/organism	Application	Reference	
Type III secretion systems						
*Escherichia coli* K12	T3SS of EPEC	Tir	Tir	HeLa cells	Protein translocation avoiding pathogenicity	(Ruano-Gallego et al., 2015)
Enteropathogenic *E. coli* (EPEC)	T3SS	CyaA	Tir, Tir_N15,_ Tir_N20,_ Tir_N26,_ Tir_N>26_ (in the absence of CesT), Tir_N>59_ (in the presence of CesT), Tir_N20_-Cif_ΔN20_	HeLa cells	Effector translocation signal and chaperone studies	(Crawford et al., 2002; Charpentier and Oswald, 2004; Munera et al., 2010)
		Single-domain antibodies (sdAbs)	EspF_1-20_	HeLa cells	Intracellular delivery of antibodies	(Blanco-Toribio et al., 2010)
EPEC and enterohemorrhagic *E. coli* (EHEC)	T3SS	β-lactamase	Cif_N16_ (and longer, until Cif_N256_) Map, Map_N20_, Map_N20_-Cif_ΔN20_ EspF_N20_, EspF_N20_-Cif_ΔN20_ EspA_N20_, EspA_N20_-Map_ΔN20_, EspA_N20_-Tir_ΔN20_ EspB_N20_, EspD_N20_	HeLa cells	Translocation signal studies	(Charpentier and Oswald, 2004; Munera et al., 2010)
*Pseudomonas aeruginosa*	T3SS	β-lactamase	ExoS_1-54_	Dendritic cells from C57BL/6 mice, HL60 cells	Generation of antitumor immune response	(Derouazi et al., 2010; Le Gouëllec et al., 2013)
		Epitopes of glioma antigens (TRP-2, gp100, Survivin, MUC18, hgp100)	ExoS_1-54_	Dendritic cells from C57BL/6 mice	Generation of antitumor immune response	(Derouazi et al., 2010)
		OVA_257-264_	ExoS_1-54_	C57BL/6J mice APCs	Generation of antitumor immune response	(Le Gouëllec et al., 2013)
		Cre recombinase	ExoS_1-54_	TE26 cells	Genome editing	(Bichsel et al., 2011)
		TALEN	ExoS_1-54_	HeLa cells, mESCs, hESCs, hiPSCs	Genome editing	(Jia et al., 2014; Jia et al., 2015)
		MyoD	ExoS_1-54_	Mouse embryonic fibroblasts	Directing cellular differentiation	(Bichsel et al., 2013)
		Oct4, Sox2 and Nanog	ExoS_1-54_	Human fibroblasts, cord blood CD34+ hematopoietic stem cells	Induction of pluripotency	(Berthoin et al., 2016)
		GATA4, MEF2c, TBX5, ESRRG, and MESP	ExoS_1-54_	mESCs, hESCs or iPSCs	Directing cellular differentiation	(Bai et al., 2015; Jin et al., 2018)
*Salmonella enterica* serovar Typhimurium	SPI1 and SPI2	CyaA	Ssph1_N140_ (SPI1 and SPI2) Ssph2_N143_ (SPI2)	HeLa cells, bone marrow-derived macrophages and RAW264.7 murine macrophages	Effector and translocation signal studies	(Miao et al., 1999; Miao and Miller, 2000)
			SlrP (SPI1 and SPI2) SseI, SseJ (SPI2) SspA (SPI1)	RAW264.7 murine macrophages	Effector and translocation signal studies	(Miao and Miller, 2000)
			SteA_N10,_ SteA_N20_ (SPI1 and SPI2)	RAW264.7 murine macrophages, HeLa cells	Effector and translocation signal studies	(Cardenal-Muñoz and Ramos-Morales, 2011)
			SseB (SPI2)	RAW264.7 murine macrophages	Translocation signals, importance of chaperones and CBDs for translocation	(Zurawski and Stein, 2003)
		β-lactamase	SptP (SPI1)	Extracellular medium	Measure titer of protein secreted and its ability to refold	(Metcalf et al., 2014)
		Gag (from HIV or SIV)	SopE_1-104_ (SPI1)	RMA cells, mice	Generation of an immune response. Adaptation of the sequence to be better unfolded	(Chen et al., 2006)
		Polypeptide of Rev, Tat and Nef (from SIV and HIV) Siv-Nef_1-264_	SopE_1-104_ (SPI1)	RMA cells	Generation of an immune response. Adaptation of the sequence to be better unfolded	(Chen et al., 2006)
		SaEsxA and SaEsxB from *S. aureus*	SipA_1-507_ (SPI1)	Macrophages, BALB/c mice	Generation of prophylactic vaccines	(Xu et al., 2018)
		PcrV from *P. aeruginosa*	SseJ	Macrophages, C57BL/6 mice	Generation of prophylactic vaccines	(Aguilera-Herce et al., 2019)
		Influenza nucleoprotein (IVNP_366-374_)	SptP (protein inserted in nt 285)	RMA cells, C57BL/6J mice	Generation of an immune response	(Rüssmann et al., 1998)
		Murine lymphocytic choriomeningitis virus nucleoprotein (LCMVNP_118-126_)	SptP (protein inserted in nt 285)	BALB/c mice	Generation of an immune response	(Rüssmann et al., 1998)
		p60 from *Listeria monocytogenes*	SspH2 (SPI2) SifA (SPI2) SopE2 (SPI2)	Macrophages BALB/c mice	Generation of an immune response, immunoprophylaxis of tumors	(Panthel et al., 2008)
	SPI1 in minicells	Ovoalbumin (OVA)	SopE_1-104_	RMA cells, dendritic cells	Generation of an immune response	(Carleton et al., 2013)
*Yersinia enterocolitica*	T3SS	CyaA	YopE_N130_	HeLa cells	Effector and translocation signal studies	(Sory and Cornelis, 1994)
		Alkaline phosphatase	YopH_1-48_ (or longer to increase efficiency)	Extracellular medium	Protein translocation and translocation signal studies	(Michiels and Cornelis, 1991)
		α-peptide of β-galactosidase	YopH_1-48_ (or longer to increase efficiency)	Extracellular medium	Protein translocation and translocation signal studies	(Michiels and Cornelis, 1991)
		CRA (*Trypanosoma cruzi* protein)	YopE	Extracellular medium	Generation of an immune response in mice	(Sory et al., 1992)
		Cytoplasmic neomycin phosphotransferase	YopE_1-15_ YopN_1-15_	Extracellular medium	Secretion signal characterization	(Anderson and Schneewind, 1997)
		p60 from *Listeria monocytogenes*	YopE_1-18_, YopE_1-53_, YopE_1-138_ Deletions and insertion of the antigen in the YopE sequence	Murine P388D_1_ cells, HEp-2 cells	Generation of an immune response	(Rüssmann et al., 2000; Igwe et al., 2002)
		IpgB_1_ and IpgB_2_ from *Shigella* Map from *E. coli*	YopE_1-53_, YopE_1-138_	HeLa cells	Study of cellular functions of effectors	(Wölke and Heesemann, 2012)
		Truncated H3 interacting-domain (tBID)	YopE_1-138_	HeLa cells	Study of cellular functions of effectors	(Ittig et al., 2015)
*Yersinia pestis*	T3SS	β-lactamase	YopE	HeLa cells	Testing for inhibition of translocation by chemical compounds	(Pan et al., 2009)
*Yersinia pseudotuberculosis*	T3SS	LLO from *L. monocytogenes*	YopE_1-18_, YopE_1-138_	P388D_1_ cells, macrophages	Generation of an immune response	(Igwe et al., 2002; Rüssmann et al., 2003)
Type IV secretion systems						
*Agrobacterium tumefaciens*	VirB/D4	Cre recombinase	VirF, VirF_C37_, VirF_C19_ VirE2 VirE2_C50_ VirE3_C50_ VirD4*^Mesorhizobium loti ^* Msi061 *^Mesorhizobium loti ^* Msi059 *^Mesorhizobium loti ^*	*Arabidopsis thaliana, Saccharomyces cerevisiae*	Protein translocation and translocation signal studies	(Vergunst et al., 2000; Schrammeijer et al., 2003; Vergunst et al., 2003; Vergunst et al., 2005)
			VirD2 VirD5_C50_ MobA_C48_	*Arabidopsis thaliana*	Protein translocation and translocation signal studies	(Vergunst et al., 2005)
		I-SceI	VirD2	YPH250 and RSY12 yeast	Genome modification	(Rolloos et al., 2015)
		Cas9	VirD2	*Nicotiana benthamiana* *Saccharomyces cerevisiae*	Genome modification	(Schmitz et al., 2020)
*Bartonella henselae*	VirB/D4	TrwC relaxase + DNA	BID, *	EA.hy926, HeLa cells	Translocation of a relaxase-DNA complex through a pT4SS	(Fernández-González et al., 2011; Gonzalez-Prieto et al., 2017)
		MobA relaxase +DNA	*	EA.hy926, HeLa cells	Translocation of a relaxase-DNA complex through a pT4SS	(Guzmán-Herrador et al., 2017)
		Mob relaxase + DNA	BID	EA.hy926	Translocation of a relaxase-DNA complex through a pT4SS	(Schröder et al., 2011; Gonzalez-Prieto et al., 2017; Guzmán-Herrador et al., 2017)
*Coxiella burnetii*	Dot/Icm	β-lactamase	CBU1825_C50_	THP-1 cells	Effector and translocation signal studies	(Chen et al., 2010)
		TrwC relaxase + DNA	BID	HeLa cells	Translocation of a relaxase-DNA complex through a pT4SS	(Fernández-González et al., 2011; Gonzalez-Prieto et al., 2017)
		MobA relaxase +DNA	*	HeLa cells	Translocation of a relaxase-DNA complex through a pT4SS	(Guzmán-Herrador et al., 2017)
*Helicobacter pylori*	Cag	β-lactamase	CagA	AGS cells	Effector and translocation signal studies	(Schindele et al., 2016)
		HiBit (11-residue split luciferase)	CagA	AGS cells	Effector and translocation signal studies	(Lettl et al., 2021)
*Legionella pneumophila*	Dot/Icm	CyaA	LepA LepB	J774 macrophage	Effector identification	(Chen et al., 2004)
		β-lactamase	33 *Legionella* effectors	J774 Cells	Effector identification	(De Felipe et al., 2008)
			164 *Legionella* effectors (Lpg) Lpg2844_C100_ LepA	U937 macrophages	Effector identification	(Zhu et al, 2011; Allombert et al., 2017)
			RalF	C57BL/6 macrophages, alveolar epithelial cells, dendritic cells	Infection studies	(Copenhaver et al., 2014)
			CBU1825_C50_	THP-1 cells	Effector and translocation signal studies	(Chen et al., 2010)
		TrwC relaxase + DNA	RalF_C20_	CHO FcγRII	Translocation of a relaxase-DNA complex through a pT4SS	(Guzmán-Herrador et al., 2017)
		MobA relaxase + DNA	*	CHO FcγRII	Translocation of a relaxase-DNA complex through a pT4SS	(Guzmán-Herrador et al., 2017)
Type VI secretion systems						
*Pseudomonas aeruginosa*	T6SS	β-lactamase	TplE	HeLa cells	Effector and translocation signal studies	(Jiang et al., 2016)
			PldA and PldB	HeLa cells	Study of effector activity	(Jiang et al., 2014)
*Vibrio cholerae*	T6SS	β-lactamase	VgrG-1ΔACD	J774 Macrophages	Protein translocation and translocation signal studies	(Ma et al., 2009)

*These susbtrates can be heterologously delivered by the T4SS without the need of additional sequences.

Calmodulin-dependent adenylate cyclase (CyaA) is one of the proteins that have been widely used in translocation assays, and was, in fact, the first heterologous substrate whose delivery was proven, using the T3SS from *Y. enterocolitica* (Sory and Cornelis, 1994). In this work, the 130 N-terminal amino acids of the effector YopE were fused to the reporter enzyme CyaA. The fusion protein was translocated through the T3SS from *Y. enterocolitica* into HeLa cells, and the activity of the reporter enzyme was analyzed by measuring cAMP levels in the target cell. In a similar work, it was reported that CyaA could be translocated to eukaryotic cells fusing it to the 140 and 143 N-terminal residues of Ssph1 and Ssph2 *S.* Typhimurium effectors, respectively, through any of its two different T3SS (Miao et al., 1999; Miao and Miller, 2000). They also showed the importance of some of the conserved residues of the secretion signal, and they identified new effectors by generating CyaA fusion proteins with their N-terminal sequences and detecting translocation (Miao and Miller, 2000). Also using CyaA fusions, the secretion signal has been narrowed down to 10 N-terminal amino acids in the effector SteA from *S.* Typhimurium (Cardenal-Muñoz and Ramos-Morales, 2011). However, in other cases, the N-terminal secretion signal is not sufficient, and the presence of the CBD may be necessary for translocation to occur. Since it is not always located at the N-terminus, sometimes the entire sequence of an effector must be fused to the heterologous substrate for it to be translocated. This effect was studied by constructing a SseB-CyaA fusion. SseB is a *S.* Typhimurium translocon protein, which needs the chaperone SseA to be exported (Zurawski and Stein, 2003).

β-lactamase is another reporter enzyme whose activity can be measured by an enzymatic assay which can be coupled to fluorescence emission. For instance, it has been fused to YopE effector from *Y. pestis* and the fusion protein could be correctly delivered to HeLa cells, allowing subsequent tests of inhibition of translocation by different compounds (Pan et al., 2009). This strategy was also used to analyze the secretion signals of multiple EPEC and EHEC effectors, narrowing them down to 16-20 N-terminal amino acids (Charpentier and Oswald, 2004; Munera et al., 2010). β-lactamase activity has also been used to measure the titer of protein secreted into the extracellular medium by *S.* Typhimurium SPI1 T3SS, using as TS the one of SptP (Metcalf et al., 2014).

Translocation of reporters fused to effectors or TS is a valuable tool to study the secretion process itself. In addition, *Y. enterocolitica* T3SS has been widely used to enhance translocation of known effectors into target cells and study their cellular functions. It is the case of *Shigella* IpgB_1_ and IpgB_2_ and *E. coli* Map, which have been researched thanks to the development of fusion proteins with *Yersinia* YopE TS, allowing the study of their activity (Wölke and Heesemann, 2012). The same strategy was used to study the proapoptotic activity of truncated H3 interacting-domain (tBID) in HeLa cells (Ittig et al., 2015).There are examples of other proteins which have been translocated fusing to them the N-terminal domain of *Yersinia* Yop effectors. The 48 N-terminal amino acids of YopH were fused to the alkaline phosphatase or the cytoplasmic peptide of β-galactosidase, reporters which allowed to detect secretion (Michiels and Cornelis, 1991). The fusion between YopE and CRA, a *Trypanosoma cruzi* specific cytoplasmic protein, could be secreted to the extracellular medium (Sory et al., 1992), as well as cytoplasmic neomycin phosphotransferase (Npt) fused to the 15 N-terminal amino acids of YopE or YopN (Anderson and Schneewind, 1997).

Interestingly, the hypothesis that the proteins are secreted directly unfolded with their N-terminus first was proven generating a fusion protein between a *S. flexneri* T3SS substrate, IpaB, and a bulky protein with a knotted motif, which cannot be unfolded. This heterologous substrate therefore obstructed the channel and allowed its visualization within the NC (Dohlich et al., 2014).

### 4.2 Translocation through T4SS

T4SS have also been used to translocate heterologous proteins by fusing reporter proteins to full length effector proteins or to translocation tags. Conjugative relaxases have been also used to translocate other fusion proteins, with or without covalently attached DNA molecules, to target cells (**Figure 1**; **Table 1**).

Several reports have shown the translocation of the site-specific Cre recombinase through the T4SS of *A. tumefaciens* VirB/D4 using the CRAfT assay. This assay has been widely used to study the translocation of effectors and TS, and it allows the measurement of the Cre recombinase activity on the *loxP* cassette, which has to be previously engineered in the recipient cell. In these works, Cre was fused to VirF, VirE2, VirE3, VirD5 and VirD2 and translocated to plant cells from *Arabidopsis thaliana* (Vergunst et al., 2000; Vergunst et al., 2003, Vergunst et al., 2005). They also showed that only by adding a tag containing the 37 C-terminal residues from VirF or 50 C-terminal residues from VirE2 and VirE3, the fusion proteins translocation could be detected (Vergunst et al., 2000; Vergunst et al., 2003). This tag was later reduced to the 19 C-terminal residues in VirF (Vergunst et al., 2005). In this work they also showed that a Cre fusion containing the 48 C-terminal amino acids of the relaxase MobA could be translocated into plant cells. Cre recombinase has also been translocated into yeast through *A. tumefaciens* T4SS, fused to full VirE2, VirF and VirE3 or the VirF 37 C-terminal residues (Schrammeijer et al., 2003). Hubber and colleagues were able to translocate substrates of the VirB/D4 system of the microsymbiont *Mesorhizobium loti*, VirD4 and the effectors Msi061 and Msi059, fused to the Cre recombinase through the *A. tumefaciens* VirB/D4 T4SS into *Saccharomyces cerevisiae* and into *A. thaliana* (Hubber et al., 2004). The strategy of fusing CyaA with effectors has been used to identify effectors from pathogens like *Anaplasma marginale*, translocated through the T4SS of *L. pneumophila* (Lockwood et al., 2011).

The BID signal has also been used for secretion of heterologous proteins through the *Bartonella* VirB/D4 T4SS. This tag was first used to increase the translocation efficiency of the Mob relaxase from a cryptic plasmid of *B. henselae*, which had been shown to be translocated through this T4SS, although with a very low efficiency. The addition of this signal to the relaxase increased approximately 100-fold its recruitment (Schröder et al., 2011). Simultaneously, TrwC relaxase was also shown to be naturally recruited by this T4SS. Addition of the BID tag did not increase the translocation efficiency in this case, probably due to the instability of the fusion protein (Fernández-González et al., 2011).

The Dot/Icm T4SS of *L. pneumophila* has also been shown to translocate heterologous proteins. Many effectors have been used to translocate reporter proteins (Chen et al., 2004; Campodonico et al., 2005; De Felipe et al., 2005; De Felipe et al., 2008; Zhu et al, 2011; Copenhaver et al., 2014; Allombert et al., 2017). Specifically, the C-terminal tag of the RalF effector, consisting on its 20 C-terminal residues, has been fused to the CyaA reporter (Nagai et al., 2005), or to the relaxase TrwC (Guzmán-Herrador et al., 2017), leading to their translocation into host cells. Similar works have been reported using the related Dot/Icm T4SS of *C. burnetii*, by adding the 50 C-terminal amino-acids from effector CBU1825 to the reporter β-lactamase (Chen et al., 2010). Finally, the CagA effector from *H. pylori* has been fused to reporter proteins, such as β-lactamase or split-luciferase tag, which have been successfully translocated to the target eukaryotic cells (Schindele et al., 2016; Lettl et al., 2021).

Interestingly, several conjugative relaxases can be naturally recognized and translocated trough T4SS involved in pathogenesis into eukaryotic cells, without the need of fusing a tag signal. The relaxase is translocated covalently attached to a molecule of DNA containing an *oriT* in a mechanism that is similar to bacterial conjugation; in fact, translocation of the relaxases is inferred by the expression in the target cells of reporter genes encoded in the transferred DNA. This has been demonstrated for conjugative relaxases Mob (pBGR1), TrwC (R388) and MobA (RSF1010) through the T4SS of *B. henselae, L. pneumophila* and *C. burnetii* (Fernández-González et al., 2011; Schröder et al., 2011; Guzmán-Herrador et al., 2017).

### 4.3 Translocation through T6SS

Some works have provided proof of concept that T6SS can also translocate heterologous proteins to eukaryotic cells (**Figure 1**, **Table 1** **)**. Ma and collaborators fused the full VgrG-1 effector or the N-terminus of a VgrG component from *V. cholerae* with the β-lactamase reporter, and detected the activity in macrophages (Ma et al., 2009). Similar works demonstrated β-lactamase delivery into HeLa cells when fused to several cargo effectors from *P. aeruginosa* (Jiang et al, 2014; Jiang et al., 2016). In spite of these examples, the use of T6SS to translocate heterologous proteins has not been widely exploited yet. It has been proposed as a great alternative since the evolved effectors are folded prior to their delivery. However, as they are an integral part of the spike, only a few molecules can be delivered by each system. On the other hand, cargo effectors are stored in high numbers in the tube, but there is not enough knowledge on the secretion signals to drive heterologous secretion.

### 4.4 Translocation into prokaryotic cells

In both T4SS and T6SS, the target cell can also be prokaryotic (Bleves et al, 2020). Moreover, effectors of T4SS involved in pathogenicity have been found to be also translocated between bacteria (Luo and Isberg, 2004) and some T6SS effectors can target both prokaryotic and eukaryotic cells (Berni et al., 2019). Thus, heterologous substrate translocation can also be used to reach other bacteria. This provides a simpler methodological setup to dig into the determinants of heterologous secretion.

In conjugative T4SS, relaxases have been fused to other proteins to pilot them into the recipient cell. Cre recombinase was successfully delivered to bacteria by fusing it with different relaxases such as TrwC, TraI or MobA (Luo and Isberg, 2004; Lang et al., 2010; Trokter and Waksman, 2018).

In the case of T6SS, the majority of works have been performed using bacteria as target cells. The T6SS from *V. cholerae* has been used for different approaches of heterologous protein translocation. The evolved PAAR2 protein or just its 12 C-terminal amino acids were fused to the Cre recombinase and successfully translocated to the target cells (Hersch et al., 2021). Truncated PAAR2 was also fused to TseC effector from *Aeromonas dhakensis* and efficiently translocated by this T6SS of *V. cholerae* to kill *P. aeruginosa*. In the same work, *V. cholerae* T6SS cargo effectors TseL or TseH were fused to Cre, allowing its successful translocation. The effector domain of VgrG-3 has also been replaced by a nuclease domain of a VgrG effector from *Salmonella enterica* subsp*. arizonae* and successfully translocated to a recipient bacteria (Ho et al., 2017).

*P. aeruginosa* H1-T6SS has also been used for heterologous substrate translocation. Chimeric proteins were constructed by fusing full or the C-terminus of canonical VgrG with the β-lactamase or the Hcp cargo effector Tse2. These chimeras were secreted to the media. However, none of them were able to be translocated into the target cells (Wettstadt and Filloux, 2020). This T6SS was also modified to translocate the cargo effector Tde1 from the T6SS of *A. tumefaciens*. Only when Tde1 was fused with the full VgrG1 protein, it was secreted to the media (the injection to the recipient cell was not achieved) (Wettstadt et al., 2020). Modification of canonical components of the T6SS spike has also been attempted by fusing the canonical VgrG1 with the evolved VgrG2b. As before, secretion to the media, but not injection into target cells was achieved (Wettstadt and Filloux, 2020).

Finally, the T6SS from *A. dhakensis* can be also used for the heterologous translocation of Cre when the recombinase is fused to VgrG, although no translocation was detected when it was fused to PAAR. In this case, none of the structural proteins carried a C-terminal extension (Hersch et al., 2021).

## 5 Applications of heterologous substrate translocation

The ability of T3SS, T4SS and T6SS to deliver proteins or even DNA directly into the cytoplasm of target cells, which can be eukaryotic or prokaryotic, has a high potential for biotechnological applications. The possibility of translocating heterologous proteins fused to the cognate substrates or to secretion signals opens the way for delivering a protein of interest to develop a specific action with biotechnological interest (**Figure 1**; **Table 1**). Most of the studies that exist so far use T3SS. However, advances in the knowledge of T4SS and T6SS determinants for translocation will likely lead to similar uses.

### 5.1 Antigen-antibody delivery

There are several works using T3SS to deliver antigenic peptides into target cells for vaccination. This strategy is based on the delivery of antigens into intracellular compartments in order to stimulate an immune response. Attenuated *S.* Typhimurium and *Yersinia* spp. have been widely used for this purpose.

This approach was firstly used to generate immunity against some viruses, and live attenuated *S.* Typhimurium was used to deliver the antigens. The influenza nucleoprotein (IVNP) or the murine lymphocytic choriomeningitis virus nucleoprotein (LCMVNP) were fused to the *S.* Typhimurium SptP effector, and its translocation through the T3SS succeeded in triggering a class-I restricted immune response, both *in vitro* and in mice, in which immune responses were triggered protecting the animals against lethal infections of both viruses (Rüssmann et al., 1998). A similar approach was attempted against the human immunodeficiency virus (HIV) and the closely related simian immunodeficiency virus (SIV) by fusing the Gag protein with SopE. Interestingly, the protein could not be translocated because of the stability of some of its regions, which prevented the necessary unfolding. The authors solved the problem by introducing mutations which relaxed this stability. They were also able to generate and translocate a polypeptide made of sequences of other HIV or SIV antigens (Chen et al., 2006).

The generation of prophylactic vaccines against two bacterial pathogens of increasing clinical interest, *Staphylococcus aureus* and *P. aeruginosa*, has also been attempted using avirulent *S.* Typhimurium as delivery strain. The *S. aureus* virulence factors SaEsxA and SaEsxB were fused to the TS of the effector SipA from *S.* Typhimurium and delivered through its SPI1 T3SS. As for *P. aeruginosa*, the antigen PcrV was fused to the effector SseJ. In these studies, the delivery of the antigens to eukaryotic cells was successful, again *in vitro* and *in vivo*, increasing the survival rates of mice after being challenged with the pathogens (Xu et al., 2018; Aguilera-Herce et al., 2019).

Another interesting approach is the delivery of therapeutic antibodies directly into the cytoplasm of eukaryotic cells, so that they can target intracellular antigens involved in diseases. So far, this strategy has been proved fusing the TS of EPEC and EHEC effector EspF to nanobodies (single-domain antibodies) recognizing amylase or GFP, and translocating the fusions through the T3SS of an attenuated EPEC. Both secretion into the supernatant and translocation into HeLa cells was observed, and the nanobodies maintained the capacity to bind their specific antigens after delivery (Blanco-Toribio et al., 2010). Subsequently, the same authors devised a way to overcome problems arising from the use of pathogenic bacteria as delivery mechanisms. They cloned and expressed a functional T3SS from an EPEC into a non-pathogenic *E. coli* strain, and were able to translocate effectors like Tir into HeLa cells (Ruano-Gallego et al., 2015). With a similar aim and using a novel strategy, Carleton et al. constructed minicells from *S.* Typhimurium capable of delivering heterologous proteins through functional SP1 T3SS. This design avoids the problem of using live attenuated cells but maintains their immunogenic capacity. They were able to deliver a small fragment of the OVA antigen fused to the SopE TS to murine RMA cells, eliciting an MHC class I-restricted immune response and generating CD8 responses *in vitro* and *in vivo*. In the same work, the authors stimulated dendritic cells ex vivo with a fusion of immunogenic peptides from *L. monocytogenes* antigens Listeriolysin O and p60 to SopE TS. When the stimulated cells were introduced to mice, they were able to protect the animals against the infection with the pathogen (Carleton et al., 2013).

A promising use of this protein delivery strategy is the possibility to generate immunity against tumors for the treatment of cancer, the so-called immunotherapeutic approaches, which aim to generate tumor-specific cytotoxic T-lymphocytes. *S.* Typhimurium has been used to deliver *via* T3SS the NY-ESO-1 tumor antigen for sarcoma, the tyrosinase-related protein 2 (TRP2) for melanoma, survivin for colon carcinoma and glioblastoma and hepatitis B virus x (HBx) for hepatocellular carcinoma (Nishikawa et al., 2006; Wang et al., 2008; Xiong et al., 2010; Zhu et al., 2010). *P. aeruginosa* ExoS effector has also been used to translocate tumor antigens against glioma cells and to present model epitopes like OVA_257-264_ to antigen presenting cells (APCs) that can generate specific cytotoxic T-lymphocytes (Derouazi et al., 2010; Le Gouëllec et al., 2013). Overall, these vaccines succeeded to induce a specific cytotoxic T-lymphocytes immune response in mice, and had therapeutic or protective effects against the tumors which they had been designed for.

*Y. enterocolitica* has also been proposed as a live vaccine carrier. Rüssmann et *al*. used this species to inject hybrid YopE-p60 antigenic peptides in the cytosol of HEp-2 epithelial cells through the T3SS. This antigen belongs to the intracellular pathogen *L. monocytogenes*, and using this strategy it was presented to the MHC-class I restricted pathway after its translocation to the target cells, indicating that *Y. enterocolitica* can also be used for vaccination. (Rüssmann et al., 2000). In a subsequent study, another species, *Y. pseudotuberculosis*, was used to translocate a fusion protein between YopE and listeriolysin O (LLO) from *L. monocytogenes*. It was tested in macrophages and immunized mice. Translocated antigens not only succeeded in the activation of the MHC class I-restricted immune response, like in the previous cases, but also the MHC class II-restricted antigen presentation, conferring higher protection against intracellular pathogens (Rüssmann et al., 2003). Simultaneous immunization with both chimeric antigens, YopE-LLO and YopE-p60, has also been tested, showing greater protection capacity than they possess individually (Igwe et al., 2002). The p60 protein has also been fused to SspH2, SopE2 and SifA effectors from *Salmonella*. The fusions are again capable of inducing a CD4 and CD8 T-cell response in vaccinated mice where the antigen is translocated through the T3SS. Interestingly, in this study the authors also propose the use of this kind of vaccines for the immunoprophylaxis of tumors, and tested it by orally vaccinating mice with the *Salmonella* strains they had generated, which translocate chimeric p60 antigens. Mice were subsequently injected with fibrosarcoma cells transfected with a p60 antigen epitope DNA, and a reduction or elimination of tumor growth was reported compared to the unvaccinated control group (Panthel et al., 2008).

### 5.2 Modifying the genome

Translocation of proteins in order to accomplish targeted genome editing is another interesting application of bacterial SS, which has been implemented using T3SS and T4SS. These works open an interesting avenue to explore the use of SS in order to deliver genomic modification tools to recipient eukaryotic cells without the need to express nucleases in the target cell. As previously mentioned, CRAfT assays where the Cre recombinase fused to *A. tumefaciens* VirB/D4 effectors were translocated into *A. thaliana* cells resulted in the genomic edition of the target cells containing a *loxP* cassette, although the goal was not the genomic edition itself (Vergunst et al., 2000; Vergunst et al., 2003; Vergunst et al., 2005).

Other works have addressed translocation of genome editing proteins in order to obtain targeted edition of the wild type recipient genome, without previous modifications. Effectors and TS from the VirB/D4 T4SS of *A. tumefaciens* were fused to proteins to promote targeted genome editing of the recipient eukaryotic cell. In a first work, protein VirD2 was fused with the endonuclease I-Sce, and enhanced targeted DNA integration activity was detected in the yeast recipient cells (Rolloos et al., 2015). The same group subsequently fused Cas9 endonuclease with the TS of the effector VirF, and detected Cas9-induced mutagenesis in recipient yeast expressing the guide RNA, and even plant cells, albeit at low frequency; in this case, the guide RNA was provided by the T-DNA which is also translocated *via* the T4SS (Schmitz et al., 2020).

The T3SS of *P. aeruginosa* has also been used to translocate several genome editing proteins fused to the N-terminal TS of the ExoS effector. Using this strategy, Cre recombinase fused to a nuclear localization signal has been delivered into human cells and proven to be able to mediate recombination in the nucleus (Bichsel et al., 2011). The same ExoS TS has been fused both to TALENs and to Cas9 to drive their translocation through the T3SS into different cell types. Cas9 failed to be delivered using this system (Bai et al., 2018), in contrast with the result obtained with T4SS, which may be due to the different level of unfolding required by T3SS and T4SS substrates. However, TALEN was successfully translocated into HeLa cells (Jia et al., 2014) and into mouse embryonic stem cells (mESCs), human ESCs (hESCs), and human induced pluripotent stem cells (hiPSCs) (Jia et al., 2015).

Another way to affect gene expression and cell fate without the need to edit the genome is by modulating transcription. Again, the T3SS of *P. aeruginosa* and the ExoS N-terminal TS have been used to deliver transcription factors such as MyoD, which regulates differentiation of cells into myocytes (Bichsel et al., 2013); the embryonic transcription factors Oct4, Sox2 and Nanog, which are able to induce pluripotency (Berthoin et al., 2016); or GATA4, MEF2c, TBX5, ESRRG, and MESP to differentiate mESCs, hESCs or iPSCs into cardiomyocytes (Bai et al., 2015; Jin et al., 2018).

Finally, it is worth noting that T4SS can be used not only to deliver proteins, but also DNA molecules. T4SS involved in pathogenesis can recruit conjugative relaxases and translocate them into the eukaryotic target covalently attached to a DNA molecule (Fernández-González et al., 2011; Schröder et al., 2011; Guzmán-Herrador et al., 2017). Once translocated, the DNA cargo is expressed in the eukaryotic cell. In addition, relaxases are active and some can promote integration of the transferred DNA through a mechanism that is unknown for the moment (Gonzalez-Prieto et al., 2017). Some relaxases were found to be naturally recruited by these heterologous T4SS. In other cases, the addition of a TS was required for recruitment, or increased the efficiency (Schröder et al., 2011; Guzmán-Herrador et al., 2017). The use of pathogenic T4SS to deliver DNA to target cells could be a good alternative to other existing options, such as bactofection or viral delivery (Llosa et al., 2012). In addition, the transported DNA could encode the necessary tools to accomplish targeted genetic modification in the recipients, as shown for T-DNA delivery in *A. tumefaciens* (Schmitz et al., 2020).

## 6 Discussion

Transkingdom communication, in terms of molecule crosstalk between prokaryotes and eukaryotes, reflects the complex relationships between both kinds of organisms, which cooperate and compete when they share a niche. It is intriguing that three types of nanomachines with such distant evolutionary origins such as phages, bacterial motility, or prokaryotic horizontal DNA transfer, have converged functionally into SS with the ability to inject proteins (or nucleoprotein complexes) into their target eukaryotic cells. Not surprisingly, these SS play a role in fundamental aspects of the bacterial biology. Their role in virulence has focused much of the attention because of its medical interest, but it is predicted that other functions related to the interplay between prokaryotic and eukaryotic cells cohabitating the same niche will be dependent on, or modulated by, the selective injection of macromolecules into the eukaryotic host cells.

Here, we have revised current knowledge on the process of substrate recruitment by bacterial T3SS, T4SS and T6SS, and we have focused on the secretion signals which drive recognition and translocation of substrates. This knowledge is key for understanding the biology of these systems, but it also opens up the possibility of the heterologous translocation of proteins, by fusing them to known effectors or to the secretion signals. In addition, in many cases there are other factors involved in the secretion process, and an important limitation is the fact that substrates have to be unfolded to different extents in order to be translocated. In spite of these limitations, heterologous translocation of reporter substrates has been accomplished with the three systems. These reporters are invaluable tools to study the secretion process itself and to infer the role of the translocated effectors in the cell biology of the target eukaryotic cell.

On top of this, the use of bacterial secretion systems as delivery nanomachines for customized substrates of interest is already a reality, as evidenced by the different applications reported. T3SS have been widely used in the translocation of different peptides or proteins for biomedical applications such as vaccines or combating cancer. The success on a number of approaches, which are increasingly being tested in animal models, suggests that we could envision clinical applications not far in the horizon. Both T3SS and especially T4SS have been used for the delivery of genetic modification tools directly into the target cell, allowing its modification *in vivo*. The ability of T4SS to inject a DNA molecule together with the protein adds a yet almost unexplored potential in this field. Although direct applications using T6SS as a delivery system have not been achieved yet, the proof of concept that heterologous translocation can be achieved, together with the increasing knowledge in the determinants for translocation, will probably allow a fast development of their use.

There may not be an ideal secretion system to use as a transkingdom injector. Each system shows advantages depending on the sought application. T3SS are highly specialized injectors used by intracellular pathogens to efficiently translocate effectors into human cells. Consequently, these SS are the most efficient systems translocating heterologous substrates; however, translocation of substrates of bigger size or with a low degree of unfolding could be a limitation, probably reflecting the fact that these injectors have evolved to translocate a number of cognate substrates. These limitations could be overcome by using T4SS, which show a lower requirement of unfolding of the substrate for translocation, or T6SS, which are unique in their ability to send fully folded substrates. In addition, the ability of T4SS to translocate a molecule of DNA covalently attached to a relaxase substrate is an important advantage for applications in genome editing, where the DNA template could be translocated with the editing protein. Finally, substrates translocated through T3SS and T4SS are translocated one by one; in contrast, different substrates can be transported by T6SS at the same time as they are loaded in the surface of the spike or inside the tube, opening the possibility of translocating many cargo substrates simultaneously and also sending a higher amount of substrates hosted in the lumen of the tube, once we learn how to manipulate the appropriate secretion signals.

## Author contributions

DG-H and AF-G wrote and edited the manuscript. ML revised and reviewed the manuscript. All authors contributed to the article and approved the submitted version.
